# Supplementary material for: Complexome profiling of the Chlamydomonas psb28 mutant reveals TEF5 as an early PSII assembly factor
Source: Plant Cell. 2025 Mar 17;37(6):koaf055. doi: 10.1093/plcell/koaf055 (PMC12164590; doi:10.1093/plcell/koaf055)
Supplement: koaf055_Supplementary_Data [file koaf055_supplementary_data.zip › psb28_interactive.zip!/plots/intensity/qualityPlots/peptides/PDC1_Cre07.g337650.t1.2.html]

### Cre07.g337650.t1.2

Trivial name: PDC1  
SeqCoverage [median]: 35.60  
Mass: 43.71 kDa  
  
 

| Sequence | MissedCleavage | Mass [Da] | wtIntensity | muIntensity | Score |
| --- | --- | --- | --- | --- | --- |
| EVDDAVEQAK | 0 | 1102.51 | 236.88 | 0.00 | 88.95 |
| GGSIVSLMAELMGK | 0 | 1391.72 | 0.00 | 2205.81 | 51.44 |
| GLGGSMHIYNR | 0 | 1203.58 | 0.00 | 3310.31 | 57.35 |
| GLGGSMHIYNRK | 1 | 1331.68 | 0.00 | 1259.09 | 46.35 |
| GVLPAEYHVPAFDPTYKS | 1 | 1989.98 | 0.00 | 7406.38 | 65.72 |
| LLLNNGVDPADLK | 0 | 1380.76 | 0.00 | 561.51 | 81.02 |
| LLLNNGVDPADLKK | 1 | 1508.86 | 0.00 | 2179.16 | 78.76 |
| MEIAADMMYK | 0 | 1201.52 | 0.00 | 961.42 | 76.83 |
| NMYAEPLGAGMR | 0 | 1308.60 | 0.00 | 4014.63 | 101.38 |
| RMEIAADMMYK | 1 | 1357.62 | 0.00 | 729.70 | 45.26 |
| SPNFYTR | 0 | 883.42 | 696.61 | 4392.19 | 77.28 |
| TRDEINAMR | 1 | 1104.53 | 0.00 | 1821.04 | 85.36 |
| VDGMDVLAVK | 0 | 1045.55 | 0.00 | 2275.02 | 138.98 |
| VEVMPYKVHR | 1 | 1256.67 | 0.00 | 720.72 | 46.00 |
| YHGHSMSDPGSTYR | 0 | 1593.66 | 0.00 | 1175.49 | 81.90 |
|  |  |  | 933.5 | 33012.5 |  |
